# Atypical Fracture From Bisphosphonate Use in Hypophosphatasia With Improved Bone Response to Teriparatide Therapy

**DOI:** 10.1210/jcemcr/luaf291

**Published:** 2026-01-06

**Authors:** Edwin Mora Garzon, Daniel Betancourt Zuluaga, Juan David Salazar Ospina, Sebastián López Velásquez

**Affiliations:** Division of Endocrinology and Metabolism, Department of Internal Medicine and Epidemiology, S.E.S. Hospital Universitario de Caldas, Universidad de Caldas, Manizales 170003, Colombia; Research Group on Geriatrics and Gerontology, Faculty of Health Sciences, Universidad de Caldas, Manizales 170003, Colombia; Department of Internal Medicine, Universidad de Caldas, Manizales 170003, Colombia; Medical Research Group, Faculty of Health Sciences, Universidad de Manizales 170003, Manizales, Colombia

**Keywords:** atypical fracture, bisphosphonate, adult hypophosphatasia, teriparatide, metabolic bone disorder

## Abstract

Hypophosphatasia is a rare metabolic bone disorder that is often misdiagnosed. We present the case of a middle-aged woman initially misdiagnosed with rickets and later as osteogenesis imperfecta and treated with zoledronate, after which she developed atypical femoral fractures. After switching to teriparatide therapy, her bone mineral density improved significantly. This case underscores that persistently low alkaline phosphatase with fragility or atypical fractures should prompt evaluation for hypophosphatasia and that antiresorptives (eg, bisphosphonates) may precipitate atypical fractures in this condition and should be avoided. Disease-specific therapy is enzyme replacement with asfotase alfa; anabolic therapy may improve bone mineral density in selected adults when asfotase alfa is unavailable.

## Introduction

Hypophosphatasia (HPP) is an extremely rare disease, with an estimated prevalence of 0.1 to 9 per 100 000 individuals [1]. Although it is more commonly observed in the pediatric population, there are few reports in medical literature about its presentation in adults, leading to uncertainties about the optimal treatment for this patient group. Teriparatide has been used off-label in adult patients with HPP. Limited reports indicate that this treatment appears to provide clinical and biochemical benefits, promoting gains in bone mineral density (BMD) and possibly aiding in fracture prevention [2]. We present the case of a middle-aged woman diagnosed with HPP in adulthood who, after 2 doses of zoledronic acid, experienced a fragility fracture and, following teriparatide therapy, showed significant improvement in trabecular BMD.

## Case Presentation

A 37-year-old woman was referred to the endocrinology department due to pain in the right lower limb, tooth loss, and bone deformities in the femur and tibia, with abnormal curvatures. Several clinical tests were performed, including ionized calcium, 24-hour urinary calcium, phosphorus, total proteins, albumin, 25-hydroxyvitamin D, 1,25-dihydroxyvitamin D, deoxypyridinoline cross-links, intact PTH, creatine, and complete blood count, all within normal ranges. Sequelae of rickets were diagnosed.

Additionally, a dual-energy X-ray absorptiometry (DXA) scan revealed abnormal values in the lumbar spine and femoral neck (see Table 1). Treatment with calcium and vitamin D supplements was initiated. The patient returned to the clinic at age 44, showing deterioration in DXA results. Due to radiographic findings suggestive of osteogenesis imperfecta, the diagnosis was reconsidered, and treatment with zoledronic acid was started. She received 2 doses of this medication: 1 in 2016 and another in 2018.

**Table 1.** Longitudinal BMD values and T/Z-scores measured by DXA in the lumbar spine (L1–L4), hips, and left 33% radius over time

Date	BMD L1–L4	T/Z L1–L4	BMD left total hip	T/Z left hip	BMD right total hip	T/Z right hip	BMD left 33% radius	T/Z radius
Oct. 2011	0.795 g/cm²	−2.3/−2.1	0.553	−3.1/−3.0	—	—	—	—
Sept. 2015	1.009 g/cm²	−1.4/−0.4	0.551	−3.6/−2.6	0.620 g/cm²	−3.1/−2.1	—	—
Oct. 2018	1.113 g/cm²	−0.6/+0.4	0.615	−3.1/−2.2	0.674 g/cm²	−2.6/−1.7	—	—
May 2019	1.133 g/cm²	−0.4/+0.7	—	—	0.722 g/cm²	−2.3/−1.3	—	—
Aug. 2020	1.168 g/cm²	−0.1/+0.2	—	—	—	—	0.754 g/cm²	−1.9/−1.9
Sept. 2021	1.191 g/cm²	+0.1/+0.5	—	—	—	—	0.692 g/cm²	−2.1/−2.1
Apr. 2022	1.252 g/cm²	+0.6/+1.0	—	—	—	—	0.696 g/cm²	−2.1/−2.0

The percentage change in BMD at L1-L4 between May 2019 and April 2022 was +10.5%. The change in the left 33% radius between August 2020 and April 2022 was −7.69%.

BMD is reported in g/cm² (SI: kg/m²; 1 g/cm² = 10 kg/m²).

Abbreviations: BMD, bone mineral density; DXA, dual-energy X-ray absorptiometry; L1–L4, lumbar vertebrae 1-4.

At age 47, while standing, she experienced severe pain and the inability to walk. X-rays revealed 2 fragility fractures: a displaced transverse fracture in the left femur and an incomplete fracture in the right femur, with varus deformity.

Given that these fractures occurred during bisphosphonate treatment, therapy with teriparatide (20 mcg subcutaneously daily) was initiated between 2019 and 2021, resulting in significant improvement in BMD. The results of DXA measurements before and after teriparatide treatment are summarized in Table 1.

## Diagnostic Assessment

At age 50, during a follow-up visit with endocrinology, additional clinical tests were requested. Although serum levels of calcium, phosphate, intact PTH, and 25-hydroxyvitamin D were normal, low levels of alkaline phosphatase (ALP) 10 U/L (SI: 0.17 μkat/L) (reference range, 45-115 U/L [SI: 0.75-1.92 μkat/L]) were observed. This led to the consideration of a diagnosis of hypophosphatasia, which was confirmed by genetic analysis, identifying a pathogenic variant NM_000478.6(ALPL) .892G>A p.(Glu298Lys) in the gene encoding tissue-nonspecific alkaline phosphatase (TNSALP). This variant affects a conserved residue TNSALP and was classified as pathogenic by the clinical laboratory; phenotype specificity (low ALP, fractures) supports clinical validity. Zygosity was homozygous per the accredited clinical laboratory report; parental testing was recommended.

## Treatment

Once hypophosphatasia was suspected, antiresorptive therapy was discontinued. Enzymatic replacement with asfotase alfa was indicated; however, it was not dispensed. Consequently, the patient was treated with teriparatide at 20 mcg subcutaneously once daily from 2019 to 2021.

## Outcome and Follow-up

**Figure 1.**
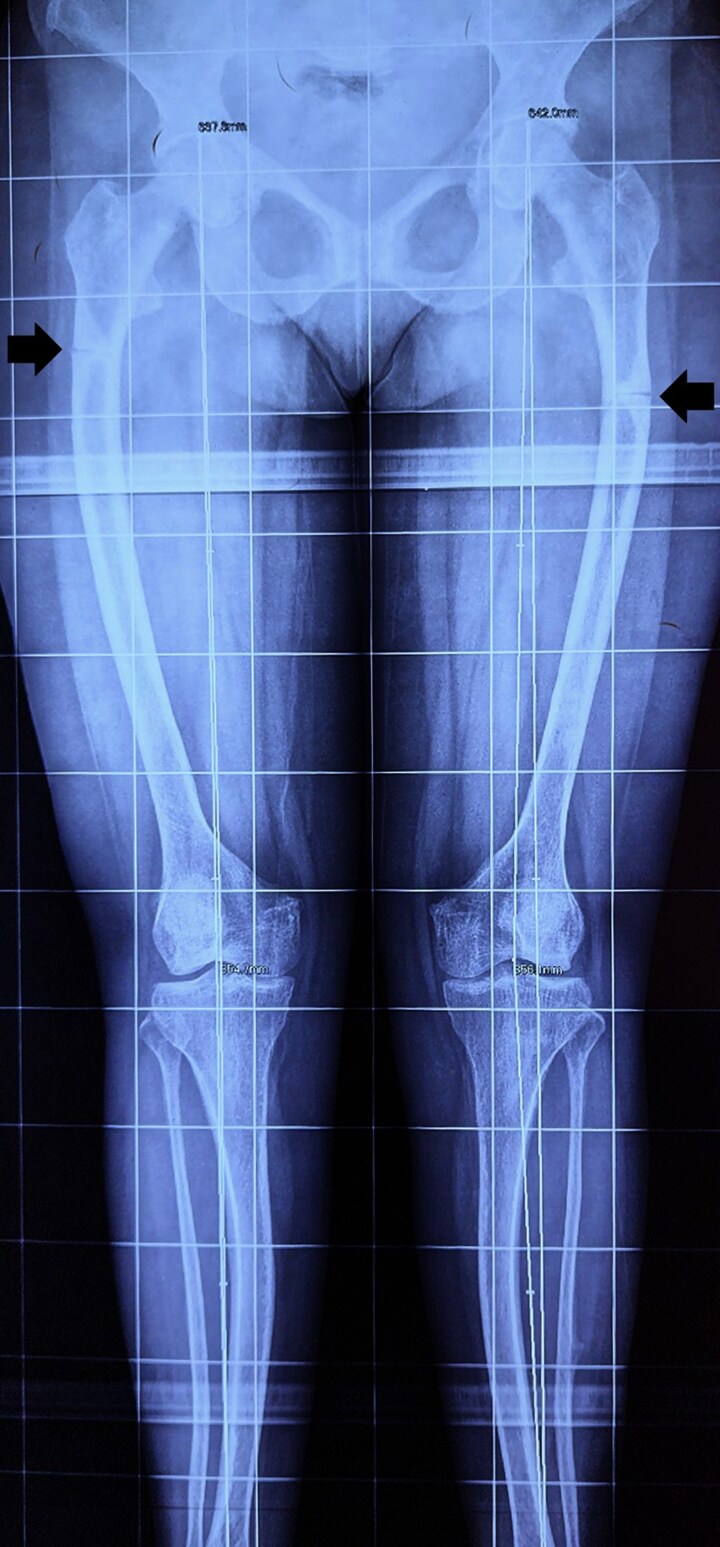
Anteroposterior radiographs of both femora at presentation. Bilateral cortical fracture with varus angulation. These findings are characteristic of atypical femoral fractures in the setting of prior bisphosphonate exposure.

**Figure 2.**
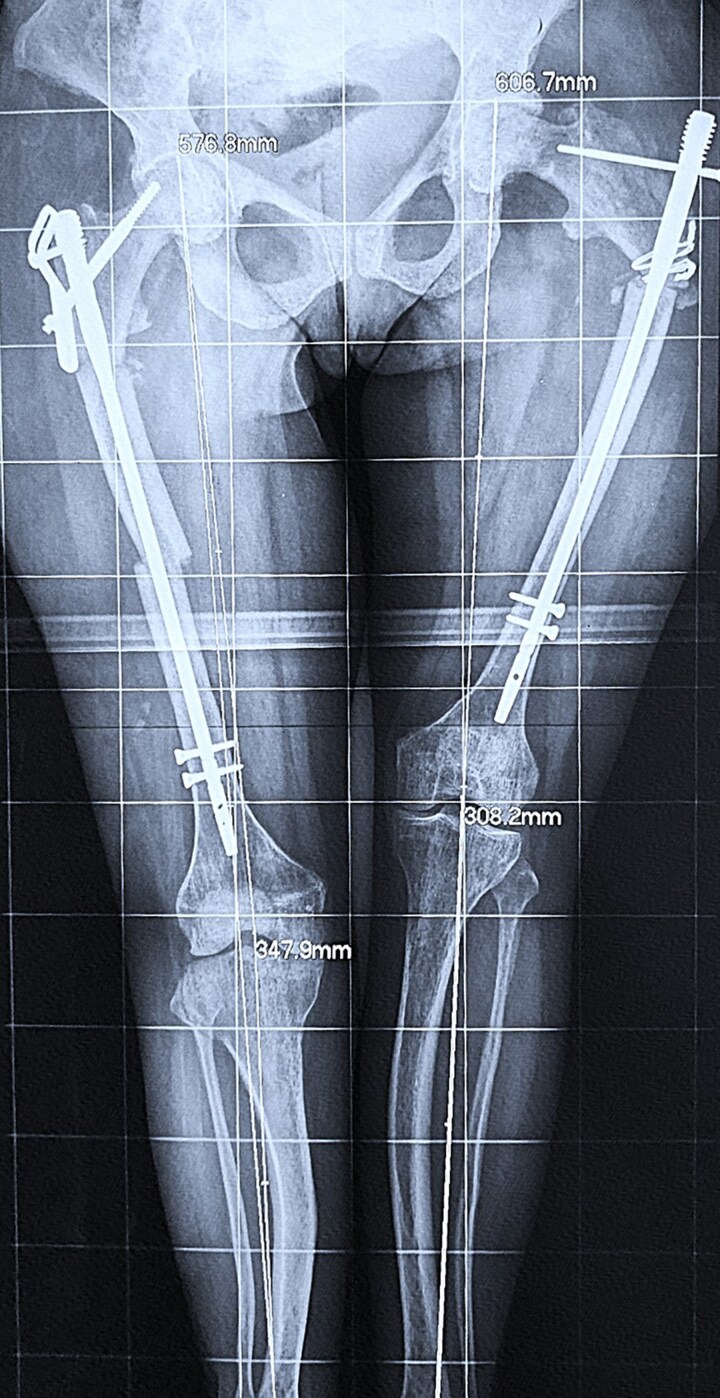
Postoperative radiographs after osteosynthesis.

During teriparatide therapy, the patient experienced functional improvement. DXA showed a + 10.5% increase in L1-L4 BMD from May 2019 to April 2022, while the one-third radius changed by −7.69% from August 2020 to April 2022; site-specific hip values are summarized in Table 1. Radiographs of atypical femoral fractures and osteosynthesis are shown in Figs. 1 and 2, respectively. Follow-up care focuses on fracture surveillance, pain control, mobility maintenance, and avoidance of antiresorptives.

## Discussion

HPP is a rare hereditary metabolic bone disease, and diagnosis is often delayed, with an average diagnostic time of 5.7 years [3]. This disease results from a congenital metabolic defect characterized by low serum ALP activity (hypophosphatasemia). This biochemical marker reflects loss-of-function mutations within the gene encoding TNSALP, affecting bones, teeth, and causing other systemic manifestations [4]. To date, more than 400 different pathogenic variants in this gene have been identified, leading to extremely variable presentations of HPP both within and between families, in children and adults, which can result in delays or even lack of diagnosis. Additionally, patients may be misdiagnosed and incorrectly treated with medications such as bisphosphonates, which can worsen the underlying defect in bone mineralization, increasing the risk of fractures [5, 6].

We present the case of a patient with a diagnostic delay of 13 years. In these scenarios, differential diagnosis can include nutritional rickets (NR), osteogenesis imperfecta (OI), and X-linked hypophosphatemia, among others. In NR and X-linked hypophosphatemia, ALP levels are usually elevated, while they are traditionally normal in OI, making ALP an initial diagnostic starting point. It is important to note that cases of HPP with normal ALP have also been reported, where the diagnostic suspicion was due to low levels of bone-specific ALP or elevated vitamin B6 levels [7]. It is also relevant to consider entities affecting ALP levels, such as liver diseases, excess vitamin D, antiresorptive therapy, hypoparathyroidism, hypothyroidism, renal osteodystrophy, and Paget's disease, among others [3]. In this case, with normal biochemical and clinical exams, the diagnosis was reconsidered from NR to OI with no measurement available as of ALP levels and was treated with bisphosphonates, a group of medications indicated in OI but which resulted in atypical femoral fractures, supporting the idea that, in HPP, bisphosphonates can be counterproductive [4].

A major limitation is the delayed recognition of HPP in our patient. Earlier testing of ALP could have prompted the diagnosis and consideration of asfotase alfa, the enzyme-replacement therapy for HPP, at a younger age.

Teriparatide may be effective in adults with HPP because intermittent PTH exerts an anabolic effect on bone, stimulating osteoblast activity and increasing bone-formation markers and bone mass. In HPP, deficient TNSALP causes extracellular pyrophosphate accumulation that impairs hydroxyapatite deposition; enhancing bone formation and remodeling can therefore facilitate mineralization and support fracture repair, even though teriparatide does not replace TNSALP [4].

Reports on teriparatide treatment are scarce but promising, although effects may vary in terms of clinical and biochemical response and may be temporary [8, 9]. Teriparatide has been used to stimulate bone formation in patients with HPP and has been observed to help improve BMD and bone quality in many cases. The response to treatment can vary between individuals, and continuous monitoring is essential to tailor treatment to the specific needs of the patient.

This case highlights the importance of accurate diagnosis in rare metabolic bone diseases like hypophosphatasia. Inadvertent use of bisphosphonates in these patients can lead to atypical fractures and worsening bone status. Teriparatide emerges as a promising therapeutic option, although more studies are needed to establish its long-term efficacy and safety in this population.

## Learning Points

- HPP is a rare hereditary metabolic bone disorder that is frequently misdiagnosed due to its nonspecific clinical presentation; clinicians should maintain a high index of suspicion, particularly when serum ALP levels are markedly low.
- Serum ALP should be included in the routine evaluation of patients with suspected osteoporosis or metabolic bone disease, as persistently low levels may indicate HPP; this is supported by current clinical guidelines, which recommend its use in the diagnostic evaluation of bone disorders.
- Bisphosphonate therapy is contraindicated in HPP, as it may worsen the underlying mineralization defect, increase fracture risk, and worsen patient outcomes.
- Teriparatide represents a promising therapeutic option for improving bone quality and mineral density in patients with HPP; however, individual response varies, and close clinical monitoring is essential.

## Contributors

All authors contributed substantially to this work. E.M.G. and D.B.Z. were involved in the patient's diagnosis and management. E.M.G., D.B.Z., J.D.S.O., and S.L.V. drafted the manuscript, critically revised it for important intellectual content, and approved the final version.

## Data Availability

Original data generated and analyzed for this case report are included in this published article. The ALPL variant reported in this case has been submitted to ClinVar (Accession SCV000034912). No raw sequencing files were generated by the authors; clinical testing was performed by an accredited clinical laboratory and summarized herein.
